# Mid-IR diffraction gratings fabricated via nanoimprint lithography for chirped pulse amplification

**DOI:** 10.1186/s11671-026-05110-7

**Published:** 2026-11-05

**Authors:** Giovanna Capraro, Danish Bashir, Michael Möller, Florian Schlachter, Jens Bolten, Thomas Graf, Marwan Abdou Ahmed, Max C. Lemme

**Affiliations:** 1 Chair of Electronic Devices, RWTH Aachen University, Otto-Blumenthal- Straße 25, 52074 Aachen, Germany; 2 AMO GmbH, Otto-Blumenthal-Straße 25, 52074 Aachen, Germany; 3 Institut für Strahlwerkzeuge (IFSW), Universität Stuttgart, Pfaffenwaldring 43, 70569 Stuttgart, Germany

## Abstract

Lasers emitting in the 2 μm spectral region have attracted considerable attention in recent years owing to their wide-ranging applications in science and industry, including the detection of atmospheric constituents like H_2_O, surgical procedures in medicine, and LIDAR technologies. In industry, they can be utilized in high-precision machining and materials engineering. For these latter purposes, a high-power laser is needed. This can be achieved by amplifying the laser pulse, typically using chirped pulse amplification (CPA). The crucial component of CPA, used for the compression and stretching steps, is the grating waveguide structure (GWS). In the present paper, we explore the fabrication of GWSs via nanoimprint lithography (NIL) for operation at a central wavelength of 2050 nm. The GWSs were integrated into the topmost hafnium oxide (HfO₂) layer of a multilayer HfO₂/SiO₂ dielectric mirror. The fabrication process comprised master wafer production via laser interference lithography, stamp fabrication, NIL patterning, and reactive-ion etching. The desired etch depth in HfO_2_ was a key challenge, which was addressed by implementing a chromium hard mask and a Taguchi experimental design. Scanning electron microscopy and atomic force microscopy revealed slight deviations from the designed GWS dimensions, mainly in the duty cycle. Consequently, the maximum diffraction efficiency was slightly blue-shifted and measured to be 97% at 2030 nm, i.e., lower than the 99% predicted theoretically. Our study demonstrates the feasibility of using NIL for large-area GWS fabrication with high diffraction efficiency.

**Supplementary Information:**

The online version contains supplementary material available at https://doi.org/10.1186/s11671-026-05110-7.

## Introduction

Mid-IR laser pulses are essential for applications in spectroscopy [1], molecular imaging [2], LIDAR technologies [3], medicine, particularly surgery [4], and other fields in science and industry [5]. Among mid-IR lasers, high-power ultrashort pulses have been intensely researched for generating supercontinuum and femtosecond tunable pulses [6]. These pulses are typically generated through amplification systems, which are most commonly based on chirped pulse amplification (CPA) [7, 8]. In the CPA, a pulse is stretched in time, amplified, and then recompressed in the time domain. Since their inception, CPA systems have required high-efficiency compressors to reduce losses. They rely on diffraction gratings as critical components for this compression step [9–11]. These diffraction gratings are formed by periodic lines with a certain linewidth (LW) and height. Initially, they were mostly made from gold [12], but gold gratings have a low diffraction efficiency (DE) and low damage resistance to high-power laser pulses, which limits their performance [13]. Multidielectric gratings (MDGs) reduce optical losses in the compression process, resulting in increased DE and higher laser-induced damage thresholds (LIDT), i.e., the threshold optical fluence where a material is damaged by a laser beam. MDGs are commonly made of multiple dielectric layers deposited on fused silica (FS) substrates that form highly reflective mirrors. Highly efficient compression, as required in CPA systems, needs large-area MDGs with precisely defined parameters. These include the duty cycle (DC = LW/Λ, where Λ is the grating period) and highly uniform groove depths across their entire area. Meeting these uniformity requirements is technologically challenging, especially for large-area compressor gratings on thick FS substrates. Among MDGs, grating waveguide structures (GWSs) have been extensively studied in recent years, especially for high-power lasers [14–22]. Differently from MDGs, the GWSs combine a multidielectric grating with a waveguide layer, placed right underneath the top-most grating layer.

Generally, GWSs are defined via lithography processes, followed by etching steps to transfer these patterns into the topmost layer of the multilayer stack. Laser interference lithography (LIL) [23] and electron beam lithography (EBL) [24] are the most commonly used lithography techniques for GWSs [11, 25, 26]. Structure transfer is typically achieved through a “dry” reactive ion etching (RIE) process. LIL and EBL provide high pattern fidelity but increase production costs as they require dedicated LIL setups or costly e-beam fabrication.

Nanoimprint lithography (NIL) has been proposed as a high-throughput, low-cost alternative to traditional lithography for reducing fabrication costs and easing production [27]. It requires a master template, which is still patterned via LIL, EBL, or other suitable techniques, and the structure is still typically dry etched into the master substrate. The large advantage is that this high-resolution master template can then be used repeatedly to create NIL stamps for patterning the final substrates before they are etched via RIE or other subsequent processes.

The feasibility of NIL for Mid-IR range photonics was demonstrated in early works as [28], who successfully patterned metallic metamaterials like fishnet structures and L-shaped resonators for wavelengths up to 5.25 μm. Recently, NIL has also emerged as a key enabler for next-generation flat optics and metasurfaces, offering significant advances in material diversity, such as nanoparticle-embedded resins, and the ability to pattern on flexible or non-planar substrates. As highlighted in the recent review by [29], NIL can now achieve pattern fidelities and optical efficiencies comparable to EBL for complex applications like achromatic metalenses and metaholograms. Recent experimental demonstrations further support the potential of NIL for complex optical devices. Polarization-insensitive metasurface holograms have been fabricated by using NIL combined with atomic layer deposition (ALD) and inverse-design optimization [30], and polarization-insensitive bifocal metalenses have been demonstrated by using NIL and ALD in the visible spectrum [31]. More recently, NIL has been used to fabricate polarization-insensitive metalenses with uniform focal arrays on 4-inch silica wafers, demonstrating the scalability of the approach for large-area and complex optical structures [32]. Once fully optimized for the desired specifications, this approach combines the ease, speed, and low cost associated with NIL with the patterning fidelity of LIL or EBL [33].

In our work, we leverage these capabilities to fabricate deep, all-dielectric Grating Waveguide Structures (GWS) on thick substrates, bridging the gap between high-end optical performance and scalable manufacturing. We report the fabrication and characterization of GWSs tailored for a wavelength of 2050 nm with NIL. The design and fabrication process represent a novel approach for GWSs based on hafnium oxide (HfO_2_) and silicon dioxide (SiO_2_) materials on thick FS substrates for CPA applications at this wavelength. These thick substrates required the development of specific RIE recipes and the addition of a hard mask layer. These specific processes were optimized and documented with scanning electron microscopy (SEM) and atomic force microscopy (AFM) images. We characterized the optical performance of the GWSs and conclude with a discussion on the validity of our NIL-based fabrication process for fabricating large-area, deep GWSs for compressors.

## Design

**Fig. 1:**
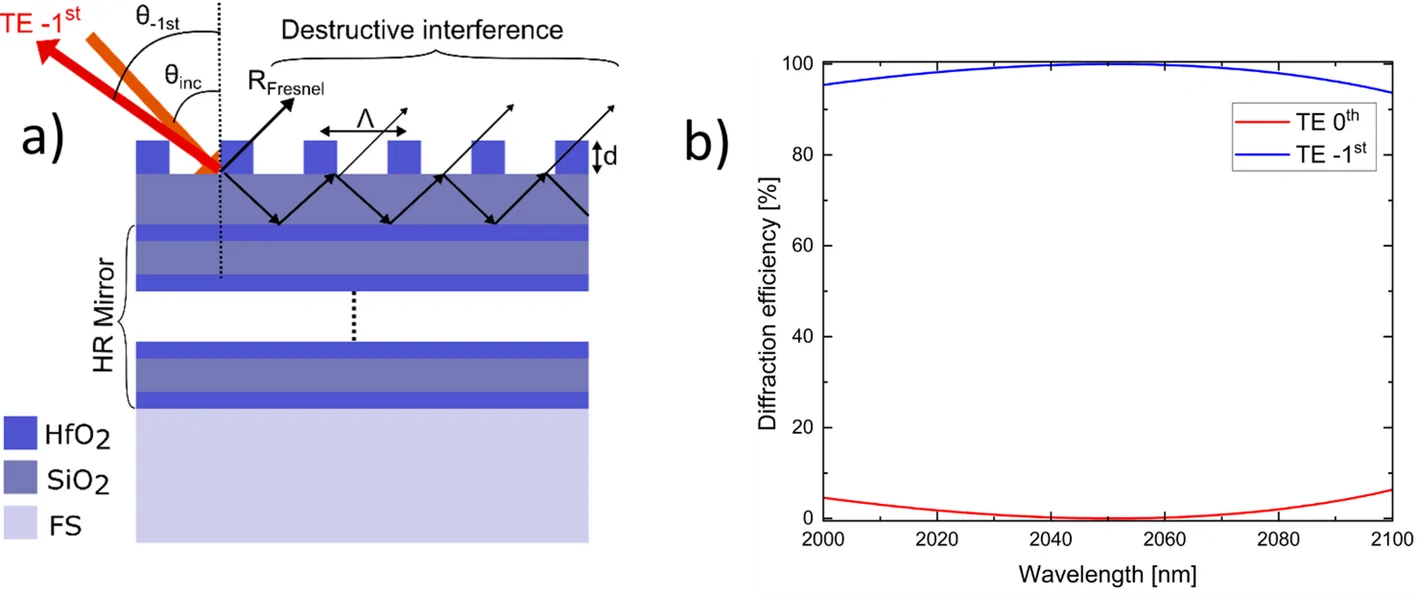
​a) Schematic of the GWS based on a resonant diffraction mechanism. The grating is etched into the top HfO_2_ layer with a period of Λ = 1190 nm, a groove depth of d = 498 nm, and a DC of 34%. ​b) Calculated spectral diffraction efficiency for TE 0th and TE -1st polarized radiation at an AOI of 55°

A resonant-diffraction GWS was designed to serve as a pulse compressor grating [19–22, 34] for use in laser architectures operating at 2 ​µm. In particular, the nominal central wavelength around 2050 nm was chosen to match holmium-doped YAG laser systems, which are commonly used in mid-IR short-pulse applications. These sources are particularly relevant for applications such as in-volume processing of silicon and polymer processing. Similar to reference [20], the GWS, shown in Fig. 1a, consists of a highly reflective (HR) multilayer mirror based on HfO₂/SiO_2_ coatings combined with a leaky-mode waveguide and a subwavelength grating.

When optimized for an off-Littrow configuration with an angle of incidence (AOI) of 55°, the GWS theoretically achieves > 99% DE over the wavelength range of 2030–2070 nm, centered at 2050 nm in the − 1st diffraction order for TE-polarized radiation. The optimized design parameters include a grating period of Λ = 1190 nm, a groove depth of d = 498 nm, and a duty cycle of DC = 34%. Figure 1b shows the calculated DE results (DE > 99% from 2030 to 2070 nm).

## Fabrication sequence

**Fig. 2:**
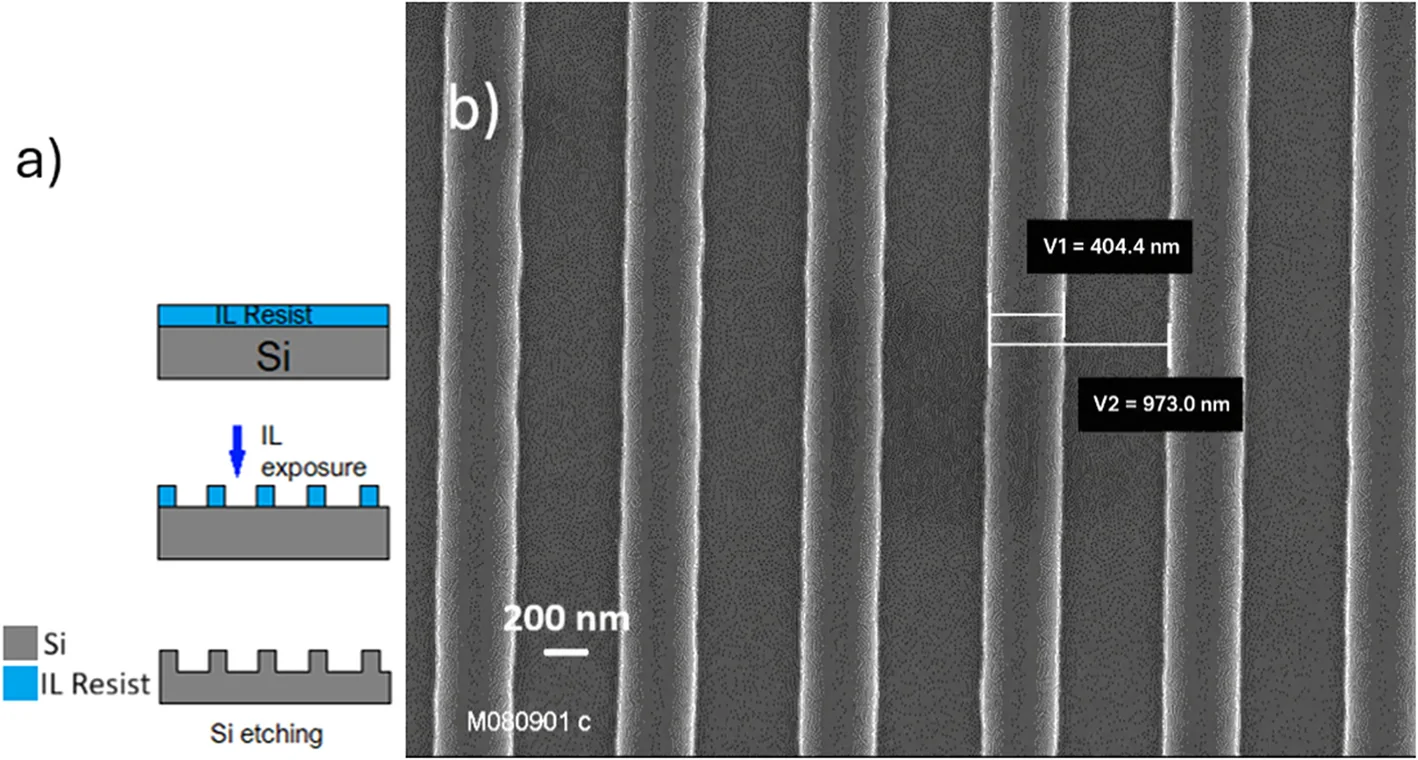
a) Scheme of the fabrication flow of the master wafer. b) SEM top view of a fabricated Si master wafer

**Fig. 3:**
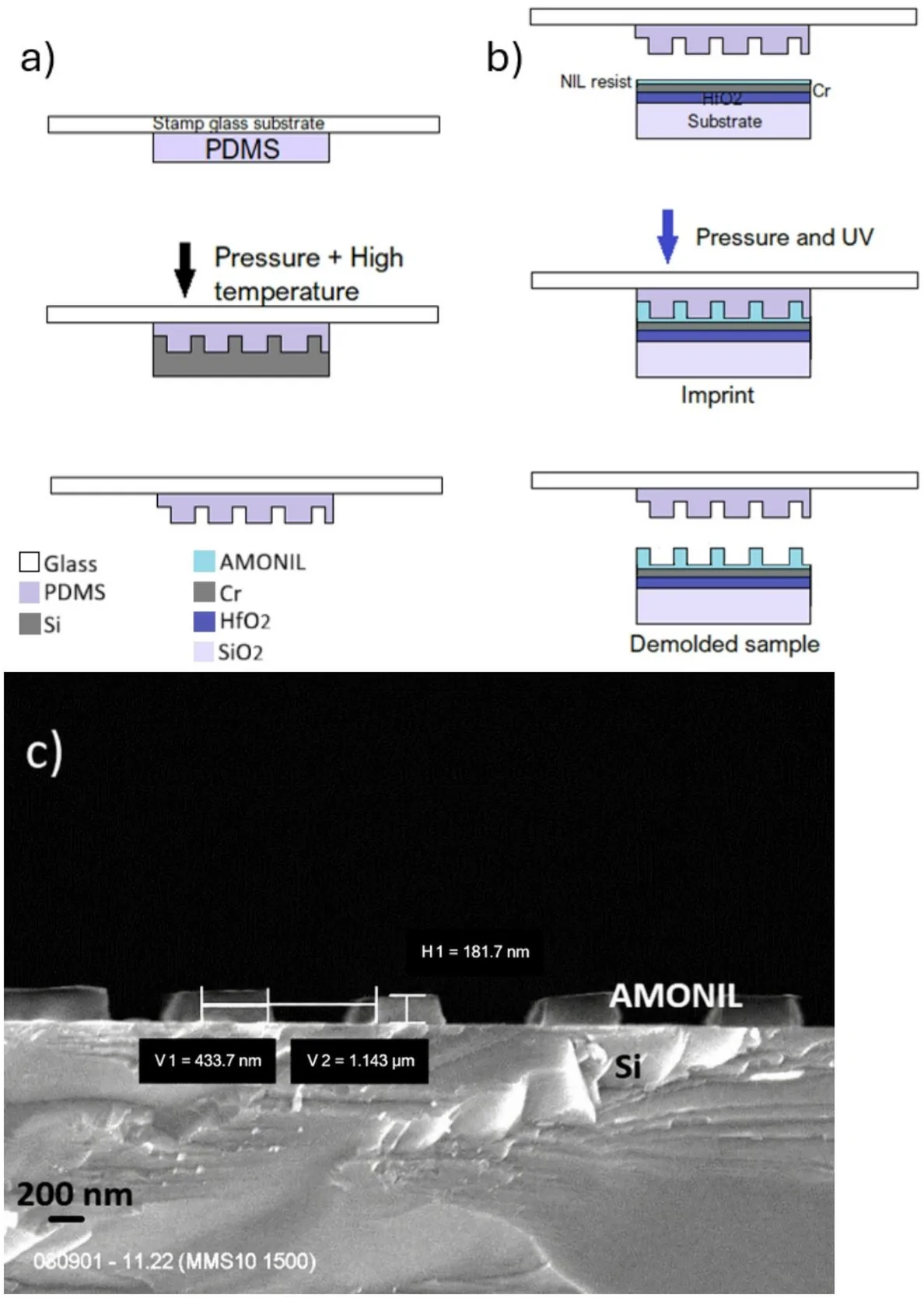
a) Fabrication flow of the stamp. b) Fabrication flow scheme of the imprint process. c) NIL on a Si test sample spin coated with the same recipe as the final substrates

**Fig. 4:**
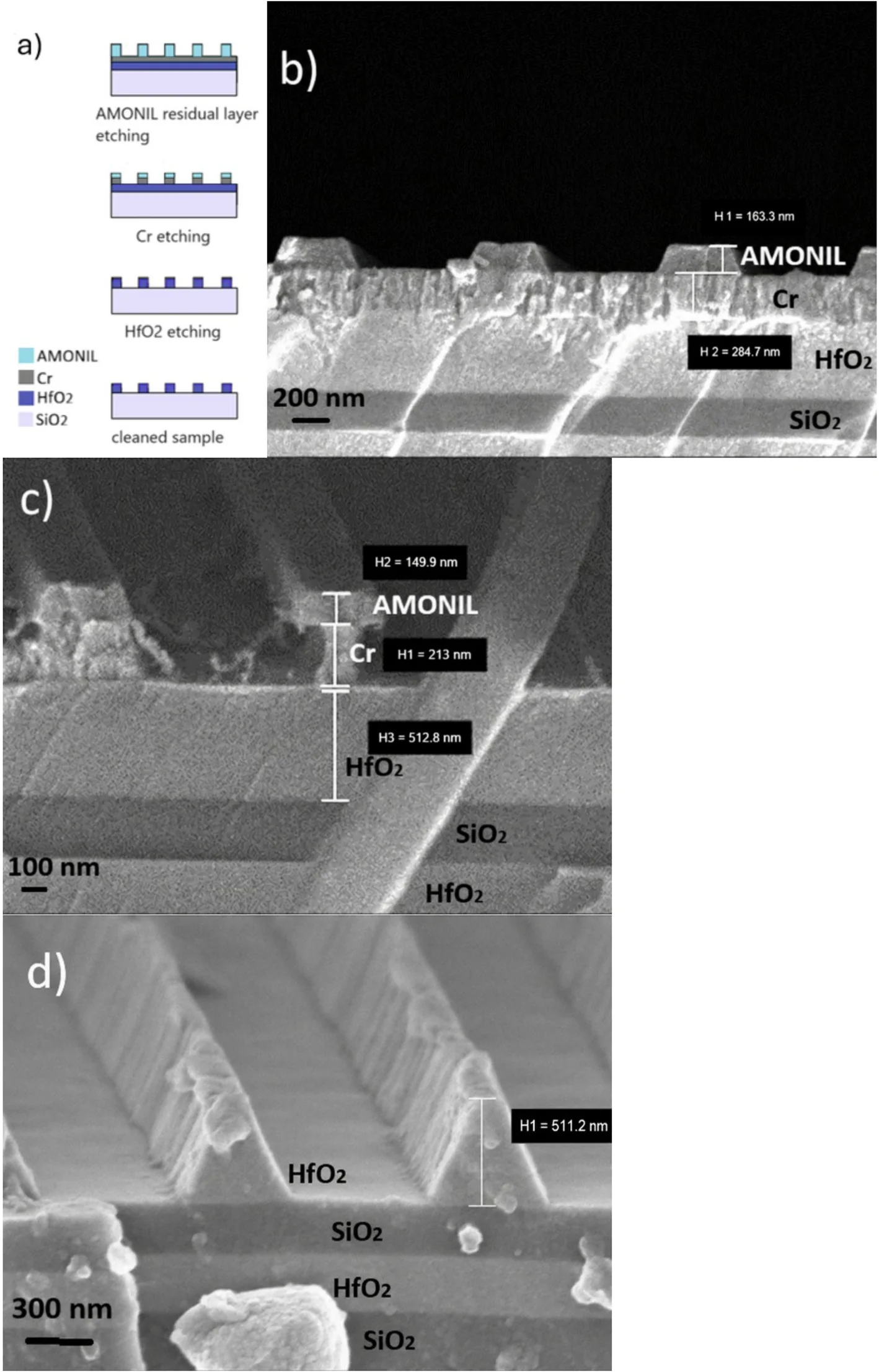
a) AMONIL residual layer, Cr hard mask and HfO_2_ grating layer etching scheme. SEM cross-sectional micrographs of the profile b) after the AMONIL residual layer etching process c) after the Cr hard mask etching process d) after the HfO_2_ etching process. b) c) and d) refer to a test sample, used as etching test during the GWSs fabrication, with the same multilayer stack as the processed substrates

The fabrication process flow can be subdivided into four major process modules: creation of a master wafer based on LIL and dry etching (Fig. 2a); creation of a stamp using this master wafer (Fig. 3a), and replication of the stamp pattern on the actual FS target substrate, previously covered with the HR multilayer and the hard mask (Fig. 3b); and etching of this pattern into the target substrate via dry etching (Fig. 4a). In particular, the etching process involves three steps: the imprint resists’ residual layer etching, the hard mask etching, and the grating layer etching.

The fabrication sequence for the GWSs starts by creating a suitable master wafer. The LIL setup used to pattern the master wafer has been developed in-house and is described in detail in [35]. It operates at a wavelength of 266 nm and is aligned based on diffraction angle measurements [36] to provide the desired grating period of 1190 nm (see Fig. 1).

A SEMI standard 150 mm silicon (Si) wafer was used as the substrate for the master. It was spin-coated with a bottom anti-reflective coating (BARC) to minimize reflections during the exposure and a positive tone DUV resist (DuPont™ UV6), for a total thickness of 300 nm. The exposure to UV light via the LIL setup and the postexposure processing of the resist mask, including development, was carried out as detailed in [35]. We then transferred the LIL pattern 190 nm deep into the Si wafer with a sulfur hexafluoride (SF_6_) based process in an Oxford Instruments Plasmalab 100 ICP-RIE tool. The etch depth was optimized to provide a sufficiently thick resist mask for the etching process following NIL, while simultaneously enabling facile stamp production (Fig. 3a) with high pattern fidelity during the NIL replication process. An SEM micrograph of one of the fabricated Si master wafers is shown in Fig. 2b. Finally, the master was passivated by exposing it to octafluorocyclobutane (C_4_F_8_) gas.

After a cleaning procedure, the master was used to produce stamps (see Fig. 3a) on 700 μm thick, 30 × 30 cm^2^ glass as a backplane. We prepared stamps formed by two polydimethylsiloxane (PDMS) materials: a high (GELEST^®^ xPDMS) and a low Young’s modulus (SYLGARD™ 184 elastomer). After curing, the stamps were demolded from the master. To minimize pattern distortion and achieve optimum pattern fidelity, the stamp fabrication and NIL process used is a variation of surface conformal imprint lithography (SCIL) [33]. Details of the stamp fabrication can be found in [33, 37].

GWSs were fabricated on an FS substrate with a diameter of 100 mm and a thickness of 10 mm. The dielectric multilayer stack, consisting of alternating HfO_2_/SiO_2_ layers, was deposited via magnetron sputtering. Details of the coating process are described in [38–40]. The top layer consisted of 498 nm of HfO_2_. The wafer was coated with AMONIL UV-curable imprint resist aided by an adhesion promoter AMOPRIME [41]. The substrate then underwent an imprint step (Fig. 3b), followed by a 10 min curing step under UV irradiation [33]. The cross-sectional SEM micrograph of the resulting resist profile on a test sample in Fig. 3c shows a 15 nm thick residual resist layer between the ridges, typical of NIL processes.

The residual layer was etched with a trifluoromethane (CHF_3_) gas-based process in an Oxford Instruments Plasmalab 100 ICP-RIE tool, with details discussed in [42]. Although this process was carried out on a thick FS substrate, no major process adaptation was needed to achieve reliable removal of the residual layer over the whole 100 mm substrate area. The resulting NIL resist profile is shown in Fig. 4b.

One of the major challenges in GWS fabrication was finding a suitable mask to etch the required grating depth of 498 nm in the HfO_2_ layer. Direct transfer of the NIL pattern was not feasible because the nanoscale dimensions of the stamp limited the NIL resist flow into stamp features, preventing a thick NIL resist mask. Also, preliminary HfO_2_ etching tests revealed that no recipe proposed in the literature gave enough selectivity between AMONIL and HfO_2_ layers.

Therefore, a chromium (Cr) hard mask was used to facilitate the transfer of the grating into the top HfO_2_ layer.

The required Cr hard mask thickness to achieve this was estimated to be 300 nm through preliminary etching tests, whereas Cr hard masks usually have thicknesses between a few nm and 100 nm [43]. Therefore, 300 nm of Cr was deposited with an e-beam evaporator (Pfeiffer 580 Classy) on top of the mirror stack prior to the NIL process step. As a consequence of the high thickness of the Cr hard mask, the etching times required to etch the hard mask were long and resulted in a distinct undercut profile of the AMONIL-hard mask stack, as shown in Fig. 4c.

Etching tests were performed by etching for 5 min 8 mm thick FS substrates covered with 300 nm Cr with an Oxford Instruments Plasmalab 100 ICP-RIE tool.

**Fig. 5:**
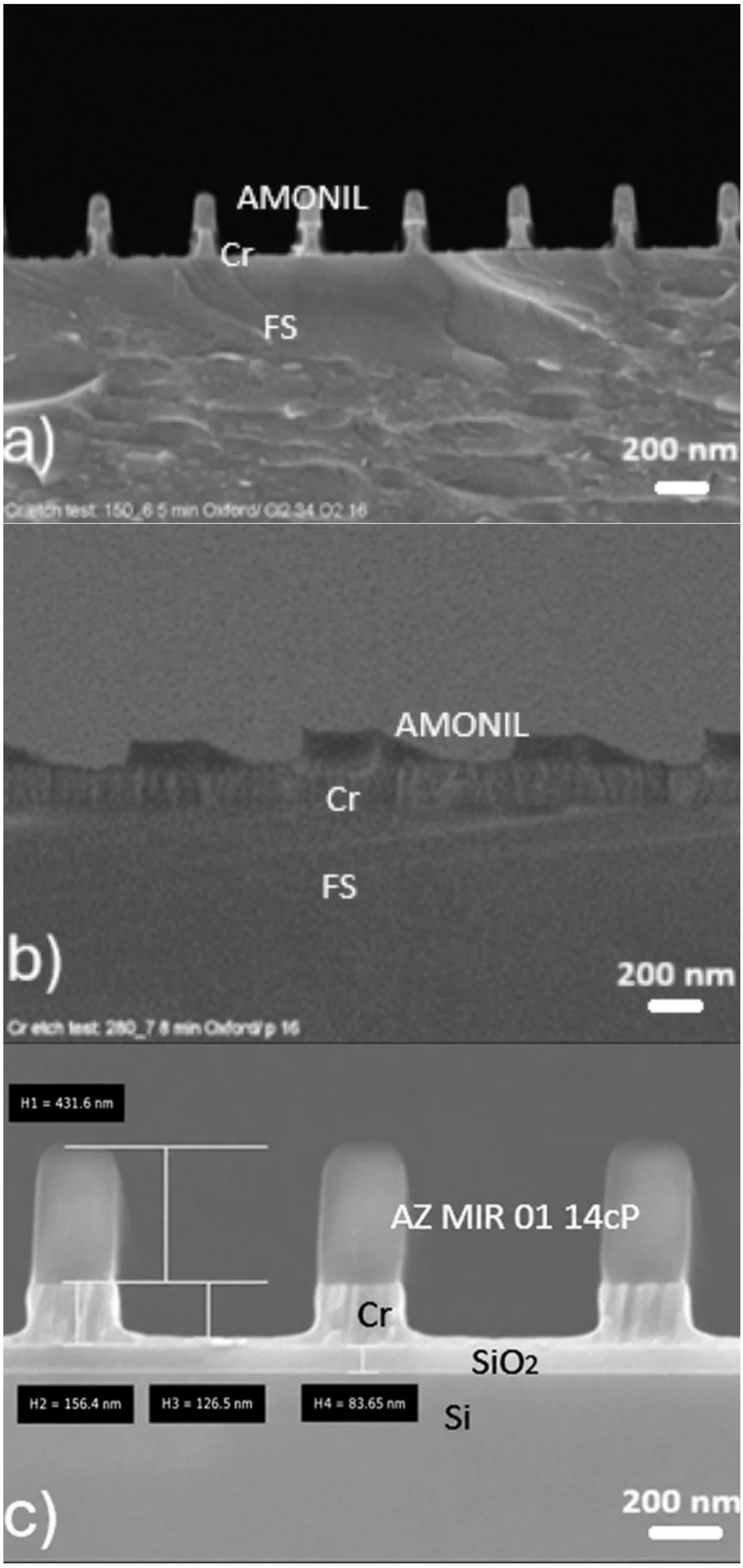
Cr etching process variation: as mentioned in Table 1, these are corresponding to a) a change in the O_2_ content and b) a change in pressure. c) Cr etching using the final optimized recipe and a different mask, AZ MIR 01 14cP (an optical lithography resist)

Starting from a Cr etching recipe based on Cl_2_/O_2_/He, the key process parameters of the etching process, the ICP and RF powers, the gas mixture composition, and the pressure, were varied in order to eliminate the undercut profile. An excerpt of the process parameters varied is in Table 1. No etching parameter set without an undercut profile could be established. Hence, we chose a recipe that minimized undercutting and maximized the etching rate while maintaining good selectivity with the AMONIL mask and preserving the mask profile. In Table 1, both the parameter space and the final recipe are summarized. This final recipe turned out to be the same as the original one. In Fig. 5a and b, we present SEM cross-section micrographs of 2 samples from these experiments (in Table 1 the process parameters regarding Fig. 5a and b are mentioned). The results of the etching tests are shown. By observing their profile, we can gain insights into the quality of the mask and Cr during etching, the resulting grating shape, and the Cr etching rate. For example, Fig. 5a shows strong mask degradation, and Fig. 5b shows that the rate was so low that there was no etching.

**Table 1:** Process variations tested for Cr etching and the optimized recipe

	ICP (W)	RF (W)	Cl_2_/O_2_ (sccm)	Pressure *p* (mTorr)
Parameter windows	1000/1100/1200	10/30/50	42/8 38/12 34/16 (34/16: Fig. 5a)	8/12/16 (16: Fig. 5b)
**Optimized final recipe**	1200	10	42/8	12

Finally, we used the selected optimized recipe to etch the same amount of Cr with another mask (AZ MIR 701 14cP) after optical lithography of grating lines with the same Λ and DC as those used for the previous tests. As shown in Fig. 5c, the undercut is much weaker than that obtained with all other recipes discussed above, which use the NIL resist mask. This finding indicates that the resist material plays a key role in the formation of the undercut, together with the etching process parameters and Cr layer thickness. Nevertheless, as our process flow included a NIL process due to the advantages mentioned in the Section Introduction, the use of an optical lithography process and therefore resist was not possible.

By carefully tuning the process parameters, a balance of the selectivity ratios between the ~ 165 nm thick AMONIL resist layer (after the residual layer was etched) and the Cr hard mask, ~ 24, as well as between the Cr and the HfO_2_ of ~ 3, was achieved. This balance allowed us to, later, fully etch the top HfO_2_ grating layer with a thickness of 498 nm on the 10 mm thick FS substrate and over its full 100 mm surface area.

The etching of the HfO_2_ grating layer also required specific process adaptations because the thick FS substrate changed the geometry and thermal conduction to the chuck in our etch chamber compared to the standard silicon wafers. We based our experiments on HfO_2_ etching recipes discussed in the literature [44–46], and chose SF_6_ as the etch gas, as it offers high selectivity for the Cr hard mask. A first experiment showed that the HfO_2_ etch rate of the initial SF_6_-based process (stated as “starting recipe” in Table 2) strongly depended on the substrate thickness and material. The HfO_2_ etch rate of approximately 200 nm/min on SEMI standard wafers became negligible 2 nm/min when applied to HfO_2_ on 5 mm thick FS substrates (all experiments were performed in an Oxford Instruments Plasmalab 100 ICP-RIE tool). Hence, we conducted a Taguchi [47] experiment to increase the HfO_2_ etch rate specifically for our 10 mm thick FS substrates while maintaining good selectivity for the Cr hard mask. Details on the Taguchi experiment are summarized in Table 2 and the SI. With the optimized recipe outlined in Table 2, we achieved an HfO_2_ etch rate of ~ 40 nm/min and a selectivity of 3 for the Cr hard mask, enabling the fabrication of functional devices with HfO_2_ structures as deep as ~ 500 nm. This process resulted in a sloped sidewall with an angle of approximately 70°, as shown in Fig. 4d. Ideally, an angle of 90° would be preferable, and may be approached with further optimizations.

**Table 2:** Excerpt of process variations tested and the final optimized process chosen for HfO_2_ etching. The tests were performed on a substrate with a thickness of 8 mm

Test	Control factors				Etch rate (nm/min)
	ICP (W)	RF (W)	SF_6_ (sccm)	Etch time (min)	
**Starting recipe**	1200	35	36	2	2
**1**	150	100	30	2	2
**2**	150	250	50	2	19
**3**	150	300	80	2	23
**4**	500	100	80	2	1
**5**	500	250	30	2	22
**6**	500	300	50	2	25
**7**	1200	100	50	2	11
**8**	1200	250	80	2	38
**9 – Optimized final recipe**	1200	300	30	2	46

**Fig. 6:**
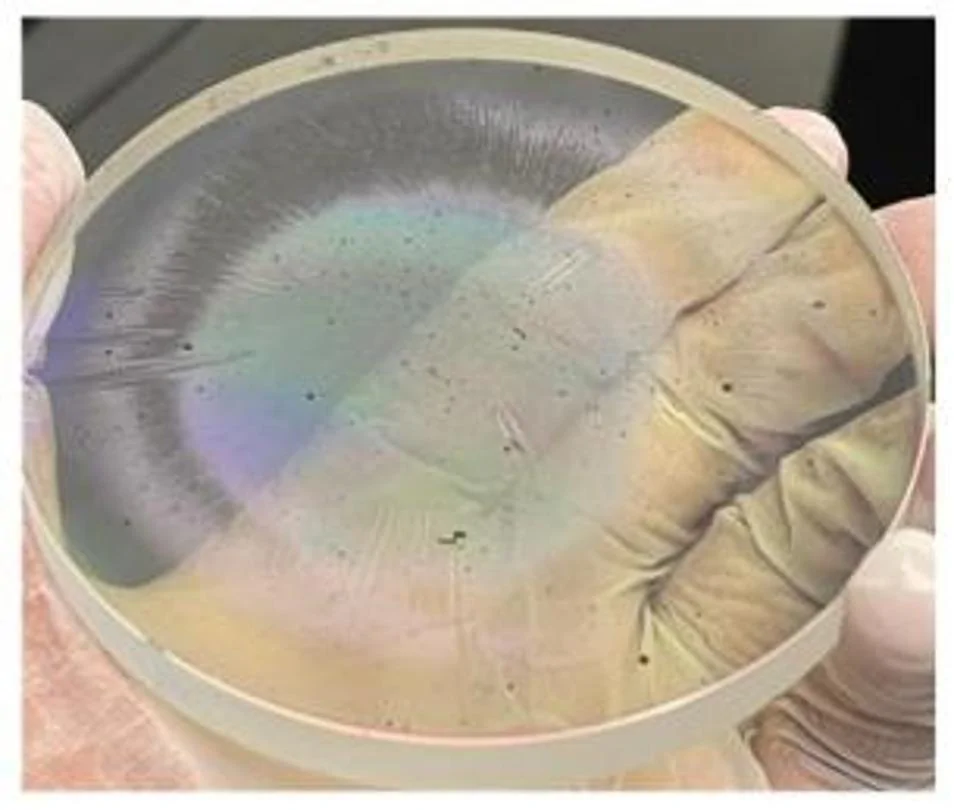
Photograph of the fabricated sample. On it, an area of approximately 5 cm diameter had a DE of at least 80%

Finally, the residual Cr mask was removed via wet chemistry. A photograph of a fabricated GWS wafer is presented in Fig. 6. Here, a circular area of approximately 5 cm radius was proved to be useful, having a DE higher than 80%. An upcoming publication by project partners with details on the DE measurements is under preparation.

## GWS SEM and AFM characterization

**Fig. 7:**
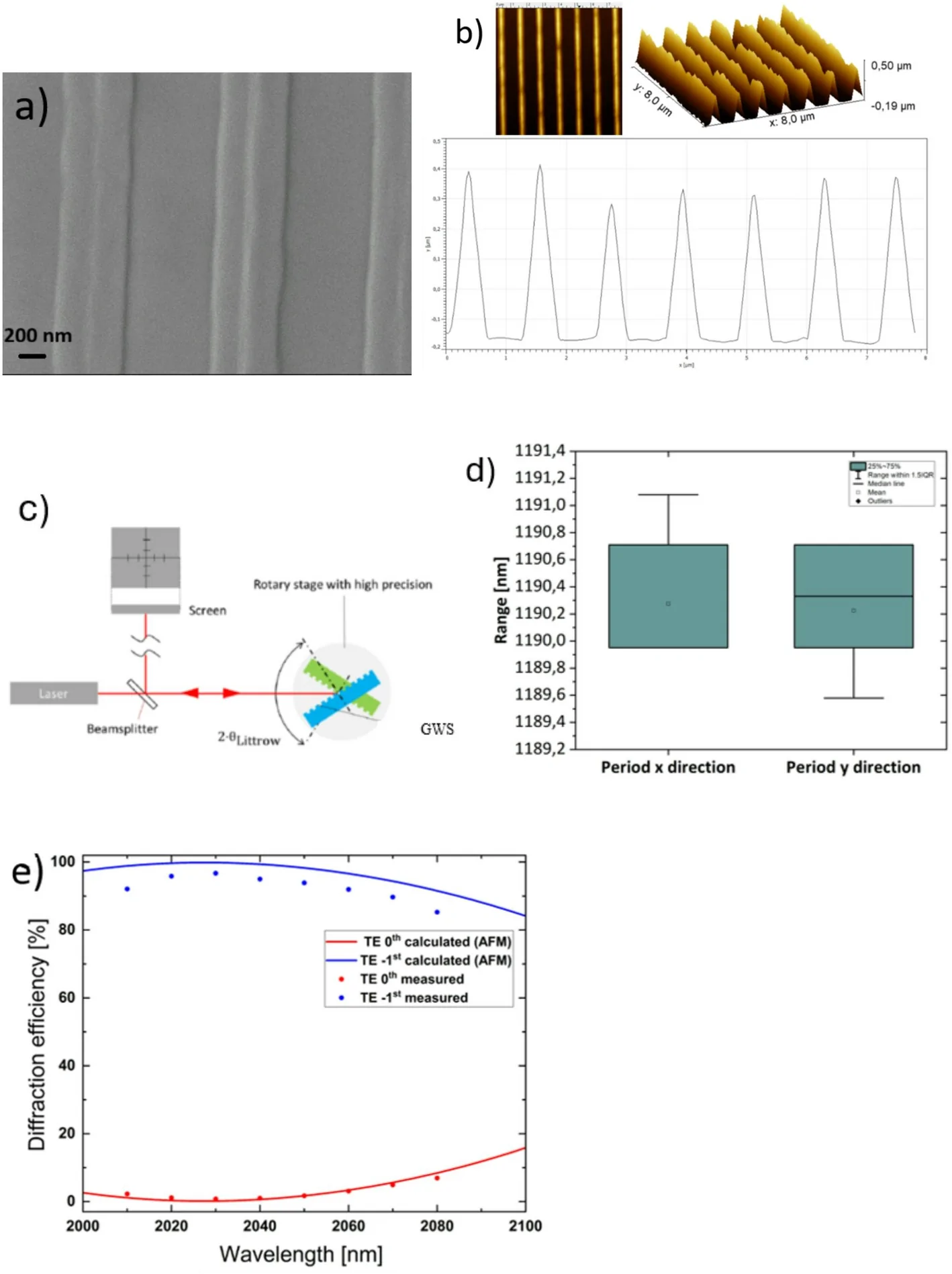
a) SEM characterization top view of the center. b) AFM image of the center. c) Scheme of the setup for period measurements. d) Box plot of the period variation along the x- and y-axes. e) measured DE data points for TE 0th and TE -1st polarized radiation at an AOI of 55°, overlaid with simulation results (lines)

The theoretical target DC of the GWS structures was 34%. The complexity of the fabrication flow necessitated by the atypical substrates and dimensions, led to detrimental effects like stamp shrinkage during curing and demolding and NIL residual layer etching, which together reduce the DC of ~ 10% on average, and undercuts during the Cr etching step, which drastically reduced further the DC by ~ 10–15% on average, respect to the master’s one. The triangular shape of the gratings after the HfO_2_ etching step improved the DC again by an average of ~ 10%. We compensated for these effects by using a Si master wafer with a DC of 42%. Nevertheless, we had a DC deviation of approximately − 7% respect to the designed target, and featured an effective DC of 28%. SEM micrographs (Fig. 7a) and AFM images (Fig. 7b) in the center of this sample confirmed a triangular profile with a sidewall angle of approximately 34° and an etch depth of about 490 nm, consistent with the data of the initial process. Furthermore, we measured the grating period along the main diameter via a Littrow-configuration-based measurement setup (Fig. 7c). The measurements revealed period variations along the x- and y-axes, with a maximum value of 1.13 nm in both directions (Fig. 7d), in line with our previous work [48]. Table 3 summarizes the effects of processing on the DC variations.

**Table 3:** DC variation over the main fabrication steps

	Master wafer	After NIL	After AMONIL residual layer etching	After Cr etching	After HfO_2_ etching (measured at 50% height)	After Cr mask cleaning (fabrication complete, measured at 50% height)
**DC (DC = LW/Λ)**	42%	38%	29%	18%	27%	28%

## Optical characterization

The DE of the fabricated sample was measured via a setup similar to that described in [49]. The AOI was set to 55°, and the sample was scanned at several positions at the central wavelength of 2050 nm to locate the spot with the highest DE. Once this spot was identified, a spectral scan was performed over a wavelength range of 2010 nm to 2080 nm.

Compared to the nominal design, the measured DE spectrum exhibits a blueshift, with a maximum DE of $\:97\pm\:0.5\%$ recorded at 2030 nm. This shift is attributed to deviations in the grating parameters of the fabricated sample, as revealed by the AFM measurements. Figure 7e shows good agreement between the measured and calculated DE spectra when the AFM-measured grating profile is taken into account in the simulation. A study on fabrication tolerances, including DC and sidewall morphology, and deviations between the original grating design and the as-fabricated structures are discussed in [50]. However, the measured DE in the − 1st order is ~ 3% lower than the calculated values, which may be mainly due to scattering and absorption losses. The AFM images in Fig. 7b and the photograph in Fig. 6 reveal surface roughness and residues on the grating at different size scales. These can scatter light, thereby reducing efficiency. Additionally, absorption within the coated layers may further contribute to the observed losses.

To better contextualize our fabrication results within the state-of-the-art of Mid-IR photonics, a detailed comparison of our process metrics and device performance with examples of other NIL-based structures reported in the literature is provided in Table 4.

**Table 4:** Comparison of this work with other NIL-based optical devices

Reference	Device Type / Application	Material	Wavelength (µm)	Key Fabrication Metric	Performance (Efficiency/Metric)
This work	GWS (CPA Lasers)	HfO_2_ / SiO_2_	2.05	Selectivity Cr/HfO_2_: ~5:1; AR ~1.5	97% DE
(Wu et al.) [28]	Metamaterials (NIM)	Au/Ag (Metals)	3.7 - 5.25	NIL + Lift-off/RIE	Neg. Index (n’ = - 1.55)
(Oh et al.) [29]	Metasurfaces (Holograms)	TiO_2_ (Resin)	Visible	Aspect Ratio up to 8.4	>90% Efficiency

## Conclusion

We demonstrated NIL-based patterning of GWSs designed for operation at 2050 nm on 10 mm-thick FS substrates with a diameter of 100 mm. The application required thick substrates, which posed severe challenges to the process flow, specifically the etching of the topmost HfO₂ mirror layer. We developed a Cr hard mask process to facilitate pattern transfer into this layer, which led to a more complex process flow. Nevertheless, a fabricated GWS, integrated within a multilayer HfO₂/SiO₂ mirror substrate, achieved a maximum DE of $\:97\pm\:0.5\%$ at 2030 nm, slightly blue-shifted from the intended 2050 nm wavelength. Although this result falls short of the state-of-the-art efficiencies achieved at the 1-micron wavelength via alternative lithographic techniques such as LIL and EBL, our GWSs show high efficiency at the 2-micron wavelength range, which has not been reported yet in the literature for CPA applications. Our work represents an important step in demonstrating the feasibility of NIL combined with dry etching for GWS fabrication for CPA application. With further optimization, efficiencies closer to the designed values appear achievable. Given the significantly lower cost of NIL than LIL and EBL, this offers a promising path for manufacturing GWSs, particularly for applications requiring a balance between optical performance and cost.

## Supplementary Information

Below is the link to the electronic supplementary material.

Supplementary Material 1

## Data Availability

The datasets generated during and/or analyzed during the current study are available from the corresponding author on reasonable request.
