# Supplementary material for: Mid-IR diffraction gratings fabricated via nanoimprint lithography for chirped pulse amplification
Source: Discov Nano. 2026 Nov 5;21(1):605. doi: 10.1186/s11671-026-05110-7 (PMC13737290; doi:10.1186/s11671-026-05110-7)
Supplement: Supplementary file 1 [file 11671_2026_5110_MOESM1_ESM.docx]

**Supporting Information for**

**"Mid-IR diffraction gratings fabricated via nanoimprint lithography for chirped pulse amplification“**

**Giovanna Capraro,^1, 2, 4^ Danish Bashir,^3, 4^ Michael Möller,^2^ Florian Schlachter,^2^ Jens Bolten,^2^ Thomas Graf,^3^ Marwan Abdou Ahmed,^3^ Max C. Lemme,^1, 2, *^**

^1^ Chair of Electronic Devices, [Faculty of Electrical Engineering and Information Technology](https://www.eld.rwth-aachen.de/go/id/mp/lidx/1/), RWTH Aachen University, Otto-Blumenthal-Straße 2, 52074 Aachen
^2^ AMO GmbH, Otto-Blumenthal-Straße 25, 52074 Aachen

^3^ Institut für Strahlwerkzeuge (IFSW), Universität Stuttgart, Pfaffenwaldring 43, 70569 Stuttgart, Germany

*lemme@amo.de

1. Taguchi experiment study for HfO_2_ dry etching process

The Taguchi experiment was performed to study the effect of etch process parameters on the HfO_2_ etch rate (R). In our experiment, three factors such as ICP power (W), RF power (W), and SF_6_ gas ratio (sccm) and levels were selected to achieve a high HfO_2_ etch rate. Based on preliminary experiment data and machine operational limitation, range and levels of the variable were selected as shown in Table SI.1.

Table SI.1: Factors and levels studied in the experiment.

| **Factor** | **ICP power (W)** | **RF power (W)** | **SF_6_ content (sccm)** |
| --- | --- | --- | --- |
| **Level 1** | 150 | 100 | 30 |
| **Level 2** | 500 | 250 | 50 |
| **Level 3** | 1200 | 300 | 80 |

The experiment was designed using an L9 orthogonal array (see Table SI.2), since it involved 3 factors with 3 levels each. The response value was, instead, the etch rate.

Table SI.2: Etching experiments performed.

| **Experiment** | **ICP power (W)** | **RF power (W)** | **SF_6_ content (sccm)** | **R (nm/min)** |
| --- | --- | --- | --- | --- |
| 1 | 150 | 100 | 30 | 2.2 |
| 2 | 150 | 250 | 50 | 18.8 |
| 3 | 150 | 300 | 80 | 22.8 |
| 4 | 500 | 100 | 80 | 1.4 |
| 5 | 500 | 250 | 30 | 22.2 |
| 6 | 500 | 300 | 50 | 25 |
| 7 | 1200 | 100 | 50 | 11.6 |
| 8 | 1200 | 250 | 80 | 37.5 |
| 9 | 1200 | 300 | 30 | 46 |

The R mean value of the three levels (calculated as the mean of the three R) was calculated for each factor. Then, delta is the value of the maximum mean minus the minimum mean. This delta value indicates an influential factor for each parameter response. Finally, the rank indicates the rank of the most influential factors to the R. Those results are in Table SI.3.

Table SI.3: Mean of each level of three factors.

| **Parameter** | **Level** | **ICP power (W)** | **RF power (W)** | **SF_6_ content (sccm)** |
| --- | --- | --- | --- | --- |
|  | 1 | 14.6 | 5.1 | 23.5 |
|  | 2 | 16.2 | 26.2 | 18.5 |
| **R (nm/min)** | 3 | 31.7 | 31.3 | 20.6 |
|  | **Delta** | 17.1 | 26.2 | 2.1 |
|  | **Rank** | 2 | 1 | 3 |

The resulting rank for R is RF power > ICP power > SF_6_ content. From the R values, the maximum of the factor RF power is at level 3 (300 W), for ICP power is at level 3 (1200 W), while for SF6 content is at level 1 (30 sccm). We expected that the RF power would be the most influential factor, since higher RF power provides a large voltage difference (higher DC bias) that accelerates positive ions and neutral species to bombard the material to be etched with high energy.

Fig. SI.1 graphically shows the results already written in Table SI.3.

Fig. SI.1: HfO_2_ etch rate trend charts of levels of factors versus parameters.

Based on the experimental results, the optimal values for three variable factors to achieve a high etch rate are RF power = 300 W; ICP power = 1200 W; and SF_6_ content = 30 sccm, which corresponds to the recipe in experiment 9. This recipe was selected for the HfO_2_ etching process.

We notice here that the etch profile was not systematically characterized alongside the etch rate. This GWS property could also affect its DE and it is an important parameter that, in future work, could be investigated.
